# Spatial and seasonal variations of isoprene secondary organic aerosol in China: Significant impact of biomass burning during winter

**DOI:** 10.1038/srep20411

**Published:** 2016-02-04

**Authors:** Xiang Ding, Quan-Fu He, Ru-Qin Shen, Qing-Qing Yu, Yu-Qing Zhang, Jin-Yuan Xin, Tian-Xue Wen, Xin-Ming Wang

**Affiliations:** 1 State Key Laboratory of Organic Geochemistry, Guangzhou Institute of Geochemistry, Chinese Academy of Sciences, Guangzhou 510640, China; 2 State Key Laboratory of Atmospheric Boundary Layer Physics and Atmospheric Chemistry, Institute of Atmospheric Physics, Chinese Academy of Sciences, Beijing 100029, China

## Abstract

Isoprene is a substantial contributor to global secondary organic aerosol (SOA). The formation of isoprene SOA (SOA_I_) is highly influenced by anthropogenic emissions. Currently, there is rare information regarding SOA_I_ in polluted regions. In this study, one-year concurrent observation of SOA_I_ tracers was undertaken at 12 sites across China for the first time. The tracers formed from the HO_2_-channel exhibited higher concentrations at rural sites, while the tracer formed from the NO/NO_2_-channel showed higher levels at urban sites. 3-Methyltetrahydrofuran-3,4-diols exhibited linear correlations with their ring-opening products, C_5_-alkenetriols. And the slopes were steeper in the southern China than the northern China, indicating stronger ring-opening reactions there. The correlation analysis of SOA_I_ tracers with the factor determining biogenic emission and the tracer of biomass burning (levoglucosan) implied that the high level of SOA_I_ during summer was controlled by biogenic emission, while the unexpected increase of SOA_I_ during winter was largely due to the elevated biomass burning emission. The estimated secondary organic carbon from isoprene (SOC_I_) exhibited the highest levels in Southwest China. The significant correlations of SOC_I_ between paired sites implied the regional impact of SOA_I_ in China. Our findings implicate that isoprene origins and SOA_I_ formation are distinctive in polluted regions.

Organic aerosol (OA) affects the earth’s radiation balance, regional air quality and public health. As a large fraction of OA, secondary organic aerosol (SOA) is produced by the reactions of volatile organic compounds (VOCs) with ozone (O_3_), hydroxyl (OH) and nitrate (NO_3_) radicals, and formed through condensation onto and/or uptake by pre-existing particles. On a global scale, isoprene (2-methyl-1,3-butadiene, C_5_H_8_) emission was estimated to be ~550 Tg yr^−1^^1^, and comprised approximately half of the annual global VOC emissions from all natural and anthropogenic sources^1^,^2^. In the future, isoprene emission would increase by more than a factor of two in year 2100^3^. Isoprene is highly reactive in the air with a SOA yield up to 28.6%^4^. The global SOA production from isoprene was estimated to be 19.2 TgC yr^−1^, accounting for ~70% of the total SOA^5^. Therefore, isoprene plays the key role in SOA study.

The formation of isoprene SOA (SOA_I_) is complex and not explicitly understood. Through the hydroperoxyl (HO_2_)-channel, isoprene reacts with the OH and HO_2_ radicals to form hydroxy hydroperoxides (ISOPOOH) which further produce isoprene epoxydiols (IEPOX) in the gas phase^6^. The reactive uptake of IEPOX by acidic particles produces SOA_I_. Under the influence of anthropogenic emissions, increasing level of nitrogen oxides (NOx = NO + NO_2_) shifts the isoprene oxidation from the HO_2_-channel to the NO/NO_2_-channel^4^. Through the latter pathway, isoprene reacts with NO and NO_2_ to form peroxymethylacrylic nitric anhydride (MPAN) which further produces hydroxymethyl-methyl-α-lactone (HMML) and methacrylic acid epoxide (MAE) in the gas phase^7^. The further reactive uptake of HMML and/or MAE by acidic particles produces SOA_I_. In the real atmosphere, the HO_2_-channel and the NO/NO_2_-channel reactions coexist and are competing. Thus, it is essential to understand the roles of these two pathways in SOA_I_ production, particularly in polluted regions.

SOA_I_ tracers can provide insight into SOA_I_ formation and spatiotemporal distribution. The identification of the HO_2_-channel products, 2-methyltetrols (2-methylthreitol and 2-methylerythritol, MTLs) revealed the importance of SOA_I_ in global SOA burden^8^. The identification of *cis*- and *trans*-3-methyltetrahydrofuran-3,4-diols (3-MeTHF-3,4-diols) discovered the acid-catalyzed intramolecular rearrangement of IEPOX in the particle phase^9^. This finding supported a previous plausible mechanism that the particulate 3-MeTHF-3,4-diols were the intermediates in the formation of C_5_-alkenetriols (*cis*-2-methyl-1,3,4-trihydroxy-1-butene, *trans*-2-methyl-1,3,4-trihydroxy-1-butene and 3-methyl-2,3,4-trihydroxy-1-butene) from IEPOX^10^. The reactive uptake of HMML and/or MAE by acidic particles produces 2-methylglyceric acid (MGA)^7^,^11^. This compound is regarded as the major tracer of SOA_I_ forming through the NO/NO_2_-channel^12^.

Large-scale and long-term survey of SOA_I_ tracers can provide a full picture of SOA_I_ fate on a continental or global scale. SOA_I_ tracers over global oceans have been measured by round-the-world cruises^13^,^14^,^15^. In the continents, Lewandowski *et al*.^16^ analyzed the characteristics of SOA tracers at 15 field sites across the United States during the spring-summer period. Our previous study made a snapshot of SOA tracers at 14 sites in China during the summer of 2012^17^. These existing large-scale observations provided unique information on SOA formation mechanisms and spatiotemporal distribution in the ambient atmosphere. However, all these studies were undertaken within one season or several months. As a major biogenic VOC, isoprene emission is driven by temperature and light^18^, and exhibits a typical seasonal trend, highest in summer and lowest in winter^3^. NOx emission is mainly driven by anthropogenic activities, and presented higher levels in winter and summer over China^19^. As mentioned above, the HO_2_-channel and the NO/NO_2_-channel reactions coexist in the atmosphere. The variations of the two pathways in SOA_I_ production depend on the relative abundances of isoprene and NOx emissions that both vary from place to place and season to season. Long-term concurrent observation over a national or continental scale is still rare and urgently needed.

China is the largest developing country and has undergone very rapid economic growth during the past decades. At present, particulate pollution is a serious environmental problem in China. The concentrations of fine particles (PM_2.5_) exceed the national air quality standards in most cities^20^. And haze events occur nationwide^21^. OA is a major component of PM_2.5_ and an important light extinction substance in China^22^. During the extremely severe haze pollution in China, SOA contributed up to 35% of PM_2.5_ and 71% of OA^23^. Thus, SOA plays an important role in particulate pollution in China. Previous modeling studies predicted that SOA was mainly from biogenic precursors in China^24^,^25^,^26^. Our ground-based observation in the 2012 summer illuminated that isoprene was the major precursor of SOA in China^17^. In this study, the observation at 12 sites across 6 regions of China extended to one year (Fig. 1). We focused on SOA_I_ tracers formed from the HO_2_-channel and the NO/NO_2_-channel, and characterized their spatial and seasonal trends. Moreover, the spatial homogeneity of the estimated secondary organic carbon from isoprene (SOC_I_) was checked between the paired sites within each region to understand the regional impact of SOA_I_.

## Results and Discussion

### Spatial distribution of SOA_I_ tracers

**Figure 2:**
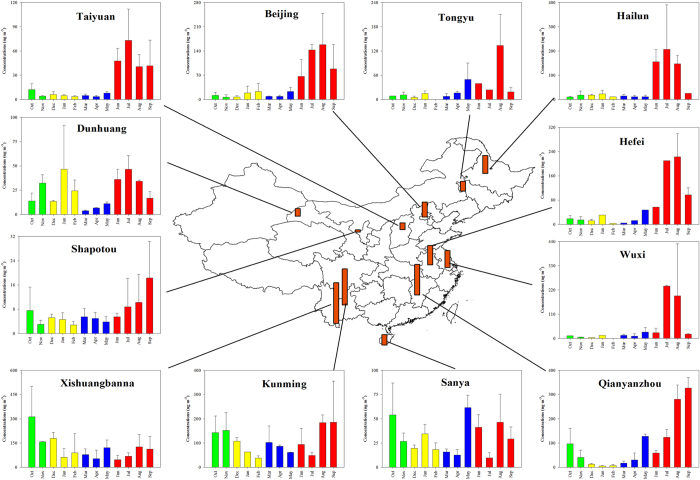
Spatial and seasonal variations of SOA_I_ tracers in China. The orange bar in the central figure represents the annual average at each site. The green, yellow, blue and red bars represent fall (October-November, 2012), winter (December 2012-February 2013), spring (March-May 2013), and summer (June-September, 2013), respectively. The figure is created by combining ArcGIS 10.1 and SigmaPlot 10.0.

The sum of the SOA_I_ tracers ranged from 6.67 to 122 ng m^−3^ among the 12 sites (Supplementary Table S1). The highest concentration was observed at the rural BN site in Southwest China, and the lowest level occurred at the desert SPT site in Northwest China (Fig. 2). Southwest China (BN and KM) exhibited the highest concentrations among the 6 regions, followed by East China (WX, HF, and QYZ), Northeast China (HL and TYU), North China (BJ and TY), South China (SY) and Northwest China (DH and SPT). MTLs were the major compounds with the annual average of 37.2 ± 28.4 ng m^−3^, followed by C_5_-alkenetriols (12.0 ± 7.69 ng m^−3^), MGA (3.66 ± 1.64 ng m^−3^), and 3-MeTHF-3,4-diols (0.36 ± 0.19 ng m^−3^).

**Figure 3:**
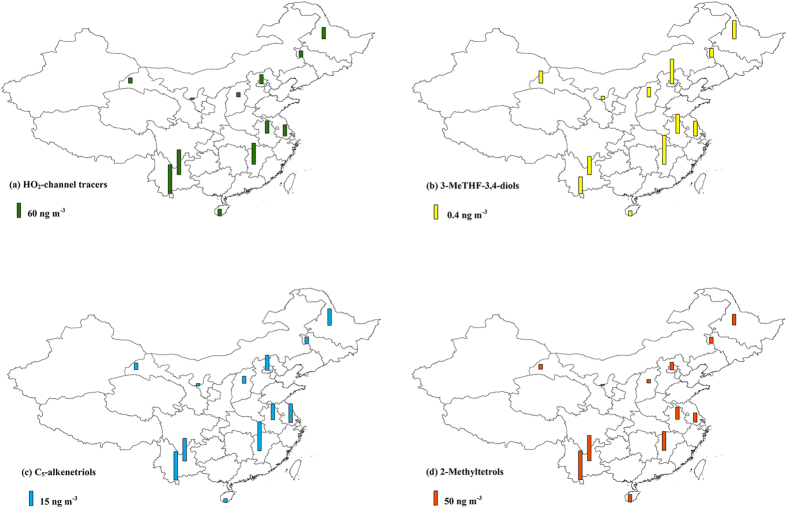
Spatial distribution of SOA_I_ tracers formed from the HO_2_-channel. The bar represents the annual average at each site. The figure is created by ArcGIS 10.1.

Figure 3 presents the spatial distribution of the SOA_I_ tracers formed from the HO_2_-channel. The highest annual average of the HO_2_-channel tracers (sum of 3-MeTHF-3,4-diols, C_5_-alkenetriols, and MTLs) was observed at the rural BN site in Southwest China (119 ng m^−3^, Fig. 3a). High concentrations were observed in Southwest China (more than 100 ng m^−3^). Northwest China exhibited the lowest levels (less than 20 ng m^−3^) compared with other regions.

3-MeTHF-3,4-diols are produced by the acid-catalyzed intermolecular rearrangement of IEPOX on acidic particles^9^. The annual average ranged from 0.08 to 0.70 ng m^−3^ with the highest level at the rural QYZ site in East China (Fig. 3b). Our measurements in the summer (0.61 ± 0.46 ng m^−3^) were one order of magnitude lower than those during summer in the southeastern United States (6.0 − 19.0 ng m^−3^)^27^,^28^. As a modeling study predicted, IEPOX concentrations in China were much lower than those in the southeastern United States (approximately 0.1 vs. 0.5 ppb) in summer^6^. Compared with the southeastern United States, China had lower isoprene emission^3^ but higher NO_x_ levels (Supplementary Fig. S1) during summer. Since IEPOX yield drops rapidly with increasing NO, high NOx levels could significantly suppress IEPOX formation^6^. Thus, relatively low isoprene emission and the suppression of IEPOX formation due to high anthropogenic emissions resulted in low 3-MeTHF-3,4-diols levels in China.

The two 3-MeTHF-3,4-diol isomers exhibited significant correlations with each other at all sites (p < 0.01, Supplementary Fig. S2), indicating that the relative formation rate of these two isomers was constant at each site. It is worth noting that the slopes of *cis*- vs. *trans*-3-MeTHF-3,4-diols exhibited a spatial difference. At the most sites, the slopes were close to 0.5. However, the slopes exceeded 1.0 in Northwest China (2.80 at DH and 1.25 at SPT). The GC-MS chromatogram also demonstrated the apparent change from *trans*- majority at SY to *cis*- majority at DH (Supplementary Fig. S3). As reported by Lin *et al*.^9^, *trans*-3-MeTHF-3,4-diol was predominant over *cis*-3-MeTHF-3,4-diol in β-IEPOX produced SOA, while *cis*-3-MeTHF-3,4-diol exhibited higher abundance than *trans*-3-MeTHF-3,4-diol in δ-IEPOX formed SOA. Paulot *et al*.^29^ pointed out that β-IEPOX accounted for ~70% of total IEPOX (β-IEPOX + δ-IEPOX) in the isoprene photo-oxidation. Thus, the dominance of *trans*-3-MeTHF-3,4-diol over *cis*-3-MeTHF-3,4-diol is expected in the ambient air, such as at the most sites in this study, and in Yorkville, southeastern United States^9^. The majority of *cis*-3-MeTHF-3,4-diol observed at DH and SPT suggested that the δ-IEPOX chemistry might play more important role in the isoprene photo-oxidation in Northwest China than other regions of China.

**Figure 4:**
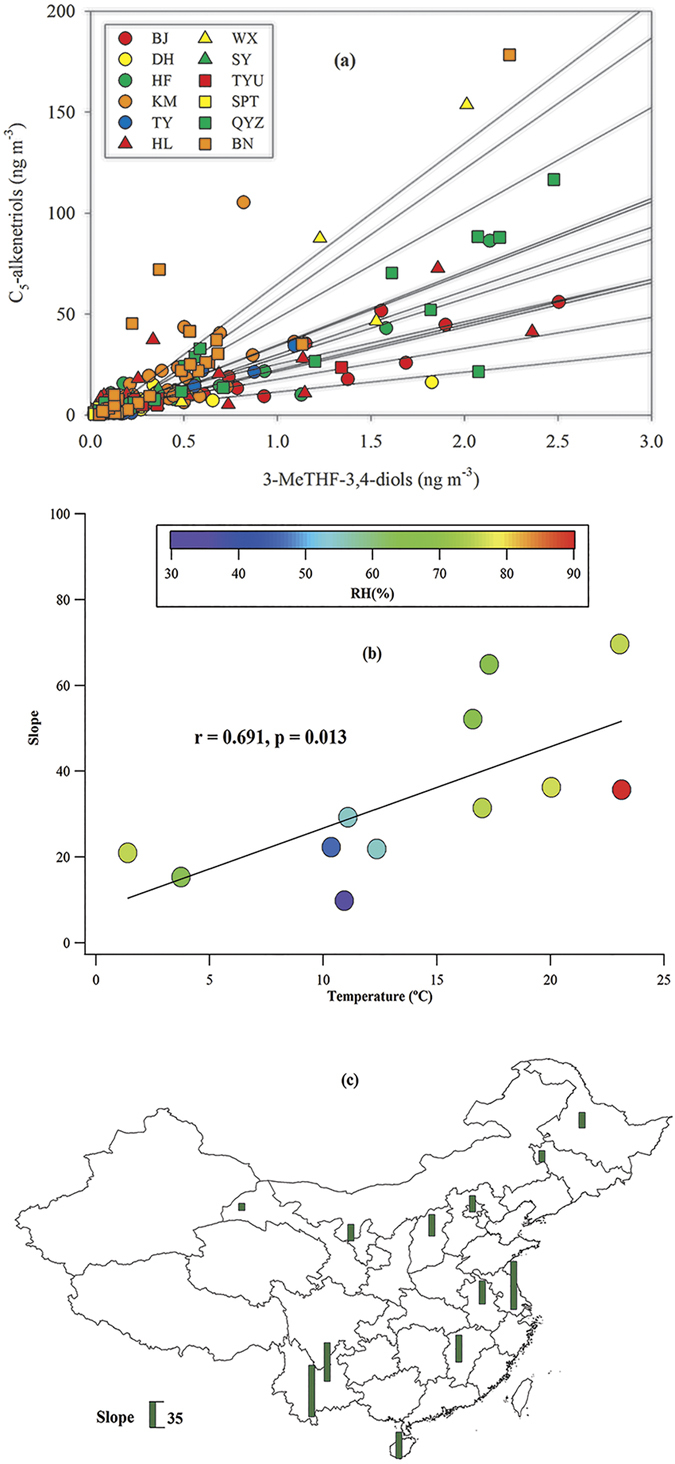
(**a**) Significant correlations between C_5_-alkenetriols and 3-MeTHF-3,4-diols at all sites (p < 0.01). Circle, triangle and square represent urban, sub-urban and rural sites, respectively. (**b**) Correlations of the slope (ΔC_5_-alkenetriols/Δ3-MeTHF-3,4-diols) with temperature and humidity. (**c**) Spatial distribution of the slope in China.

The further ring-opening reactions of 3-MeTHF-3,4-diols on acidic particles form C_5_-alkenetriols^9^,^10^. Thus, the spatial distribution of C_5_-alkenetriols (1.94 to 24.0 ng m^−3^, Fig. 3c) was similar to that of 3-MeTHF-3,4-diols, with the highest level at the rural QYZ site in East China. And C_5_-alkenetriols exhibited positive correlations with 3-MeTHF-3,4-diols at all sites (p < 0.01, Fig. 4a). Although MTLs are also formed from IEPOX^4^, the spatial distribution of MTLs was different from that of 3-MeTHF-3,4-diols and C_5_-alkenetriols. The highest annual average of MTLs occurred at the rural BN site in Southwest China (Fig. 3d). At the DH site, 3-MeTHF-3,4-diols were only correlated with C_5_-alkenetriols but not with MTLs (Fig. S4). Therefore, coming from the same precursor cannot fully explain the positive correlations between C_5_-alkenetriols and 3-MeTHF-3,4-diols at all sites.

Since C_5_-alkenetriols are the ring-opening products of 3-MeTHF-3,4-diols^9^,^10^, the increase in 3-MeTHF-3,4-diols will increase C_5_-alkenetriols. Thus, the slope of the linear correlation (ΔC_5_-alkenetriols/Δ3-MeTHF-3,4-diols) could reflect the yield of C_5_-alkenetriols from 3-MeTHF-3,4-diols. And steeper slopes suggest stronger ring-opening reactions. As temperature has positive effect on reaction rates given by Arrhenius’ equation, it is expected that the slope exhibited a positive correlation with the annual mean temperature (p = 0.013, Fig. 4b). There was no significant correlation between the slope and the annual mean humidity (p = 0.121). Higher humidity could produce more aerosol water and favor the partitioning of gas-phase species to aerosol water, as driven by Henry’s Law^30^,^31^. On the other hand, high aerosol water could dilute the concentrations of hydrogen ion, and reduce aerosol acidity, which would not favor the acid-catalyzed reactions in the particle phase^32^. Thus, we did not see a significant correlation between the slope and humidity in this study (Fig. 4b).

The slopes were steeper in the southern China (Southwest China, East China, and South China) than the northern China (Northeast China, North China and Northwest China, Fig. 4c). The broad areas in the south of Yangtze River were the acid-rain zone of China, as a result of substantial sulfur dioxide emission from large coal consumption^33^. The acidic particles over the southern China would favor the acid-catalyzed ring-opening reactions. Moreover, the high temperature in the southern China could also favor the acid-catalyzed reactions in the particle phase and enhance the conversion efficiency of C_5_-alkenetriols from 3-MeTHF-3,4-diols. Thus, it is expected that the acid-catalyzed ring-opening reactions were stronger in the southern China.

**Figure 5:**
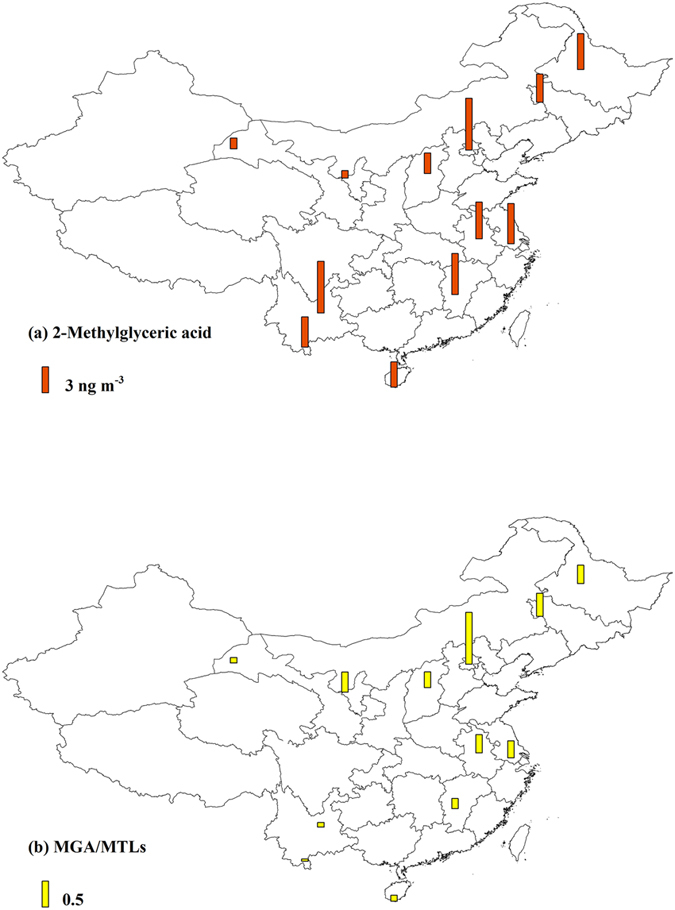
Spatial distribution of MGA and the ratio of MGA/MTLs in China. The figure is created by ArcGIS 10.1.

Increasing NOx level changes the isoprene oxidation from the HO_2_-channel to the NO/NO_2_-channel^4^. Figure 5a presents the spatial distribution of MGA, the tracer formed from the NO/NO_2_-channel^7^,^11^. Unlike the tracers formed from the HO_2_-channel, the highest level of MGA occurred at the urban BJ site in North China (annual average 5.99 ng m^−3^). And the urban sites had higher MGA concentrations than the rural sites. Since NOx emission in China was mostly from anthropogenic sources (power plants, industry and transportation)^34^, high anthropogenic emissions in urban areas would favor SOA_I_ production from the NO/NO_2_-channel. The ratio of MGA to MTLs (MGA/MTLs) can reflect the influence of NOx on SOA_I_ formation^13^. Previous chamber studies witnessed the elevated MGA/MTLs ratio with the increasing NOx/isoprene ratio^12^,^35^,^36^,^37^,^38^. The BJ site showed the highest ratio of MGA/MTLs (annual average 1.05) among the 12 sites (Fig. 5b). Previous studies combining model simulations and satellite observations demonstrated that North China was the hot spot of NOx emission in China^19^ with an annual emission trend of 4.76 ± 1.61% yr^−1^^39^. The highest MGA level and the highest MGA/MTLs ratio at the BJ site implied that the NO/NO_2_-channel had more influence on SOA_I_ formation in North China compared with the other regions.

### Seasonal variation of SOA tracers

Figure 2 presents the seasonal trend of SOA_I_ tracers. The highest concentrations existed during summer at the most sites. The elevated levels were surprisingly observed from fall to winter, especially at the DH site.

Isoprene emission rate (E_I_) depends on light and temperature^18^:


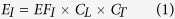


where EF_I_ is the basal emission rate at 30 °C leaf temperature and 1000 μmol m^−2^ s^−1^ photosynthetically active radiation (PAR). C_L_ and C_T_ are the factors representing the influences of light and temperature, respectively. C_L_ and C_T_ can be simply estimated as:


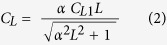



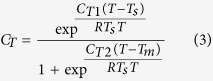


where α = 0.0027, C_L1_ = 1.066, R = 8.314 J K^−1^ mol^−1^, C_T1_ = 95000 J mol^−1^, C_T2_ = 230000 J mol^−1^, T_s_ = 303 K, T_m_ = 314 K, L is PAR (μmol m^−2^ s^−1^) and T is leaf temperature (K)^18^. In this study, daily mean temperature and maximum solar radiation during each sampling episode were downloaded from China Meteorological Data Sharing Service System (http://cdc.nmic.cn/home.do). PAR can be estimated by solar radiation multiplying photon flux efficacy of 1.86 μmol J^−1^^40^. With daily mean temperature and maximum PAR, C_T_ and C_L_ were roughly estimated for each sample. If the seasonal trend of SOA_I_ tracers is determined by biogenic emission, SOA_I_ tracers should positively correlate with biogenic emission that is controlled by C_L_ × C_T_.

As supplementary Fig. S5 a-g showed, SOA_I_ tracers exhibited positive correlations with C_L_ × C_T_ in Northeast China, North China and East China during the whole year, indicating that seasonal variation of SOA_I_ in these regions was mainly influenced by biogenic emission. However, South China, Northwest China, and Southwest China exhibited poor correlations between SOA_I_ tracers and C_L_ × C_T_ (Supplementary Fig. S5 h–l), implying that there should be other factors determining seasonal trend of SOA_I_ in these regions.

**Figure 6:**
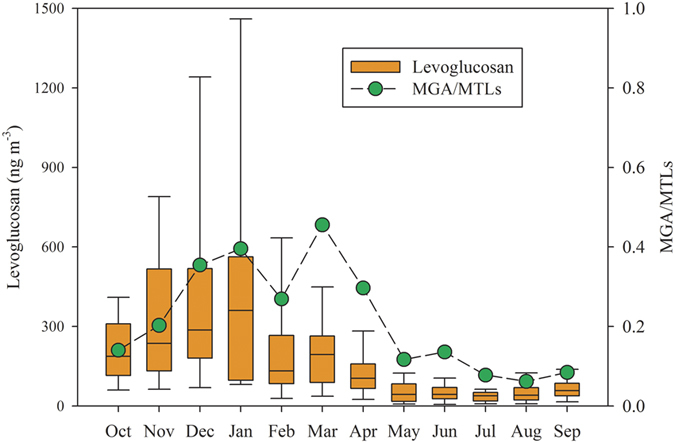
Monthly variations of levoglucosan and MGA/MTLs in China. Box with error bars represent 10^th^, 25^th^, 75^th^, 90^th^ percentiles in each month. The line in the box is the median in each month. The ratio of MGA/MTLs is the median in each month.

Figure 6 presents monthly variation of MGA/MTLs ratios in China. The ratios raised from fall to spring, and dropped in summer. As mentioned above, MGA/MTLs can reflect the influence of anthropogenic emissions (e.g. NOx) on SOA_I_ formation. The increase in MGA/MTLs in Fig. 6 implied the enhancement of anthropogenic influence on SOA_I_ formation during fall to spring. The biomass burning tracer, levoglucosan^41^ also presented higher levels from fall to spring, and dropped in summer (Fig. 6). This indicated that there was significant enhancement of biomass burning emission during fall to spring in China. Previous emission inventory studies recorded large amounts of isoprene and NOx emissions from all biomass burning types^42^,^43^. Thus, biomass burning might have significant influence on seasonal trend of SOA_I_ during fall to spring.

Considering the two facts: (1) elevated levels of SOA_I_ tracers from the fall to the spring (Fig. 2), and (2) high values of MGA/MTLs and levoglucosan during the fall to the spring (Fig. 6), we split the whole year samples into the warm period (May to September, 2013) and the cold period (October 2012 to April 2013). And the correlations of SOA_I_ tracers with the factor determining biogenic emission (C_L_ × C_T_) and the tracer of biomass burning emission (levoglucosan) were analyzed in these two periods.

As Table 1 showed, SOA_I_ tracers exhibited positive correlations with C_L_ × C_T_ but not with levoglucosan during the warm period in all regions except Southwest China. This indicated that biogenic emission was the main factor determining monthly variation of SOA_I_ during the warm period in China. During the cold period, the drop of temperature from the summer maximum to the winter minimum would lead to the biogenic emission of isoprene decreasing by 66%–99% (C_T_ drop in Table 1). And SOA_I_ tracers exhibited poor correlations with C_L_ × C_T_ during the cold period (p > 0.05) at all sites except QYZ. Thus, the unexpected increase of SOA_I_ tracers in winter could not be explained by the biogenic emission of isoprene. Differently, the enhanced biomass burning emission in the cold period would release large amounts of isoprene. And SOA_I_ tracers exhibited positive correlations with levoglucosan during the cold period (Table 1). This suggested that biomass burning emission had significant influence on monthly variation of SOA_I_ during the cold period in China. Southwest China was the exception that SOA_I_ tracers showed no dependence on biogenic emission or biomass burning emission (p > 0.05, Table 1). Biogenic emission is expected to be high all the year in Southwest China, due to high annual mean temperature and small temperature variation (e.g. 23.0 ± 2.7 °C during the whole year and 25.4 ± 0.9 °C during the warm period at BN). The spike of biomass burning emission would produce additional SOA_I_, which might compromise any significant changes in SOA_I_ levels with respect to biogenic emission in Southwest China.

### SOC estimation and correlation between sites

These SOA_I_ tracers were further applied to estimate SOC_I_ over China using the SOA-tracer method developed by Kleindienst *et al*.^44^. The researchers performed chamber experiments to obtain the mass fraction of the tracers in SOC ( *f*_SOC_) for individual precursor:


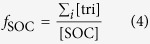


where ∑_i_[tri] is the total concentration of the tracers for a certain precursor. [SOC] is the mass concentration of SOC. With these *f*_SOC_ values and the measured SOA tracers in the ambient air, SOC from different precursors can be estimated in the atmosphere, with the assumption that the *f*_SOC_ values in the chamber are the same as those in the ambient air^44^.

For isoprene, the *f*_SOC_ was reported as 0.155 ± 0.039 μg μgC^−1^ using the total concentration of MGA and MTLs^44^. The same *f*_SOC_ and tracers were applied for SOC_I_ estimation in our previous studies in the Pearl River Delta, South China^45^, Tibetan Plateau^46^, and 14 sites across China^17^. The uncertainty in the SOA-tracer method is induced from the analysis of organic tracers and the determination of the conversion factors. The major limitation of SOA tracers is the lack of authentic standards for quantification. Instead, surrogate standards are applied to quantify SOA tracers, which induces uncertainties in the results of tracer concentrations. The errors in the tracer analyses due to surrogate quantification were within 40% for SOA_I_ tracers^46^. The uncertainty of *f*_SOC_ was reported to be 25% for isoprene^44^. Considering these factors, the uncertainty of estimated SOC_I_ was calculated as 47% through error propagation.

**Figure 7:**
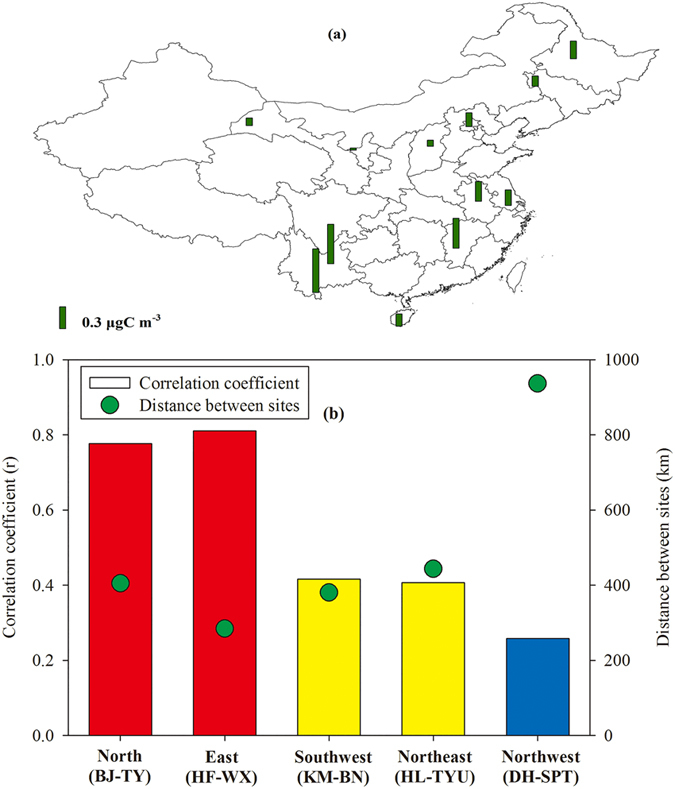
(**a**) Spatial distribution of SOC_I_ in China (**b**) Correlation coefficient of SOC_I_ between sites in each region. Red and yellow mean significant correlations at *p* < 0.01 and *p* < 0.05, respectively. Blue means poor correlation (*p* > 0.05). Figure 7a was created by ArcGIS 10.1.

Figure 7a shows spatial distribution of SOC_I_ over China. The annual average of SOC_I_ ranged from 0.03 to 0.63 μgC m^−3^ with the highest concentration at BN and the lowest level at SPT. Southwest China exhibited the highest concentrations among the 6 regions. Since there were at least two sites in each region except South China, the correlations of SOC_I_ between the paired sites in the five regions were examined to investigate whether SOC_I_ was homogenously distributed on a regional scale. Figure 7b shows the correlation coefficients (r) of SOC_I_ and the distance between the paired sites in the five regions based on the one-year data. In North China and East China, SOC_I_ presented strong correlations between the paired sites (r = 0.8, p < 0.01). The correlations within Southwest China and Northeast China were not strong but still significant (r = 0.4, p < 0.05). Since the distance between the two sites within these regions was about 400 km, the significant correlations indicated that the regional impact of SOA_I_ could be within a scale of ~400 km. When the distance between the sites increased to ~1000 km in Northwest China, the poor correlation implied the lack of large spatial homogeneity for SOA_I_. The SOA_I_ formation depends on isoprene emission, oxidant concentrations, gas- and particle-phase reactions, partitioning, and meteorology. The above factors within a certain scale, such as ~400 km would tend to be similar. However, it might not be true for a large scale, e.g., the DH and SPT pair.

## Methods

### Field sampling

Sampling was simultaneously undertaken at 12 sites in 6 regions of China, including five urban sites, three sub-urban sites, and four rural sites (Supplementary Table S2). Size-segregated particle samples were collected using Anderson 9-stage cascade impactors equipped with pre-baked quartz fiber filter (Whatman, 450 °C for 8 h) at an airflow rate of 28.3 L min^-1^. The airflow was calibrated before and after sampling using an airflow meter to keep the sampler working at the specified flow rate. One set of 9 size-fractionated filters were collected for 48 hours every two weeks at each site. In this study, 294 sets of field samples were collected from October, 2012 to September 2013. Additionally, one set of field blank were collected at each site as the same way as ambient samples for 5 minutes when the sampler was turned off.

### Chemical analysis

Each set of nine filters were combined together as one sample to meet analysis requirement. The detailed information of SOA tracer analysis is described elsewhere^17^,^46^. Isotopic labeled levoglucosan (^13^C_6_−levoglucosan) was spiked into samples as the internal standard for SOA_I_ tracers’ quantification^47^,^48^. Then, samples were extracted twice by sonication with the mixed solvent of dichloride methane (DCM)/hexane (1:1, v/v), followed by three times with the mixed solvent of DCM/methanol (1:1, v/v). The extracts of each sample were combined, filtered and concentrated to ~2 mL. Then each sample was separated into two parts. One was blown to dryness for silylation with 100 μL of pyridine and 200 μL of N,O-bis-(trimethylsilyl)-trifluoroacetamide (BSTFA) plus 1% trimethylchlorosilane (TMCS) in an oven at 70 °C for one hour. The silylated extract was analyzed for SOA_I_ tracers.

Samples were analyzed by an Agilent 7890/5975C gas chromatography/mass spectrometer detector (GC/MS) in the selected ion monitoring (SIM) mode with a 30 m HP-5 MS capillary column (i.d.0.25 mm, 0.25 μm film thickness). Splitless injection of a 2 μL sample was performed. The GC temperature was initiated at 65 °C (held for 2 min) and increased to 290 °C at 5 °C min^−1^ then held for 20 min. Eight SOA_I_ tracers were quantified, including *cis*-3-methyltetrahydrofuran-3,4-diol, *trans*-3-methyltetrahydrofuran-3,4-diol, 2-methylthreitol, 2-methylerythritol, 2-methylglyceric acid, *cis*-2-methyl-1,3,4-trihydroxy-1-butene, *trans*-2-methyl-1,3,4-trihydroxy-1-butene and 3-methyl-2,3,4-trihydroxy-1-butene. Due to lack of authentic standards, SOA_I_ tracers were quantified using erythritol^47^,^48^.

### Quality assurance/Quality control

Field and laboratory blanks were extracted and analyzed in the same way as field samples. The target SOA tracers were not detected in the field and laboratory blanks. The detection limit and recovery of erythritol were 0.04 ng m^−3^ and 80 ± 9%, respectively. Using the empirical approach developed by Stone *et al*.^49^, the uncertainties due to surrogate quantification were 35%, 15%, 20% and 85% for 3-MeTHF-3,4-diols, MTLs, MGA, and C_5_-alkenetriols, respectively. The relative differences for target compounds in paired duplicate samples (n = 6) were all <15%.

## Additional Information

**How to cite this article**: Ding, X. *et al*. Spatial and seasonal variations of isoprene secondary organic aerosol in China: Significant impact of biomass burning during winter. *Sci. Rep.* **6**, 20411; doi: 10.1038/srep20411 (2016).

## Supplementary Material

Supplementary Information

## Figures and Tables

**Figure 1:**
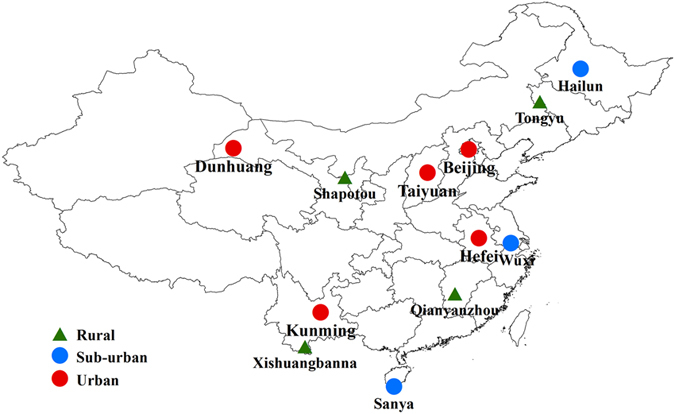
Sampling sites in China, including five urban sites: Beijing (BJ), Taiyuan (TY), Hefei (HF), Kunming (KM), and Dunhuang (DH), three sub-urban sites: Hailun (HL), Wuxi (WX), and Sanya (SY), four rural sites: Tongyu (TYU), Shapotou (SPT), Qianyanzhou (QYZ) and Xishuangbanna (BN). The figure is created by ArcGIS 10.1.

**Table 1:** Correlation coefficients of SOA_I_ tracers with biogenic emission (C_L_ × C_T_) and levoglucosan (Levo)

Region	Site	Warm period (May-September)		Cold period (October-April)		C_T_ drop^b^
		C_L_ × C_T_	Levo	C_L_ × C_T_	Levo	
Northeast China	HL	**0.803**^a^	−0.351	−0.213	**0.799**	99.9%
	TYU	**0.921**	−0.379	0.217	**0.799**	99.9%
North China	BJ	**0.796**	−0.032	−0.003	**0.694**	99.5%
	TY	**0.603**	0.347	0.101	**0.621**	99.3%
Northwest China	DH	**0.827**	0.154	−0.423	**0.734**	99.6%
	SPT	**0.844**	0.499	0.207	**0.874**	99.1%
East China	HF	**0.973**	−0.612	−0.002	**0.780**	98.5%
	WX	**0.888**	−0.384	0.458	**0.668**	98.7%
	QYZ	**0.638**	0.526	**0.663**	−0.202	98.0%
Southwest China	KM	0.005	0.586	0.159	0.169	84.0%
	BN	0.391	0.561	0.287	−0.017	66.2%
South China	SY	**0.768**	0.364	0.100	0.421	70.3%

^a^Numbers in bold are indicative of *p* < 0.05.

^b^C_T_ drop is percentage of C_T_ decrease from summer maximum temperature to winter minimum temperature using equation (3).
