# Supplementary material for: Spatial and seasonal variations of isoprene secondary organic aerosol in China: Significant impact of biomass burning during winter
Source: Sci Rep. 2016 Feb 4;6:20411. doi: 10.1038/srep20411 (PMC4740749; doi:10.1038/srep20411)
Supplement: Supplementary Information [file srep20411-s1.doc]

**Supplementary Information**

**Spatial and seasonal variations of isoprene secondary organic aerosol in China: Significant impact of biomass burning during winter**

Xiang Ding ^1^, Quan-Fu He ^1^, Ru-Qin Shen ^1^, Qing-Qing Yu ^1^, Yu-Qing Zhang ^1^, Jin-Yuan Xin ^2^, Tian-Xue Wen ^2^, Xin-Ming Wang ^1,*^

^1^ State Key Laboratory of Organic Geochemistry, Guangzhou Institute of Geochemistry, Chinese Academy of Sciences, Guangzhou 510640, China

^2^ State Key Laboratory of Atmospheric Boundary Layer Physics and Atmospheric Chemistry, Institute of Atmospheric Physics, Chinese Academy of Sciences, Beijing 100029, China

**Table S1 Summary of SOA tracers at 12 sites in China (ng m^-3^)**

|  | **Northeast China** | | | | | | | | |  | **North China** | | | | | | | | |  | **Northwest China** | | | | | | | | |
| --- | --- | --- | --- | --- | --- | --- | --- | --- | --- | --- | --- | --- | --- | --- | --- | --- | --- | --- | --- | --- | --- | --- | --- | --- | --- | --- | --- | --- | --- |
|  | **Hailun (HL)** ^a^ | | | |  | **Tongyu (TYU)** | | | |  | **Beijing (BJ)** | | | |  | **Taiyuan (TY)** | | | |  | **Dunhuang (DH)** | | | |  | **Shapotou (SPT)** | | | |
|  | Sub-urban | | | |  | Rural | | | |  | Urban | | | |  | Urban | | | |  | Urban | | | |  | Rural | | | |
|  | mean | median | min | max |  | mean | median | min | max |  | mean | median | min | max |  | mean | median | min | max |  | mean | median | min | max |  | mean | median | min | max |
|  | **Whole year (October 2012-September 2013)** | | | | | | | | | | | | | | | | | | | | | | | | | | | | |
| Temperature (°C) | 1.4 | 2.0 | -29.4 | 25.2 |  | 3.7 | 4.9 | -22.7 | 25.1 |  | 12.4 | 13.4 | -9.1 | 28.2 |  | 11.1 | 12.1 | -8.4 | 26.3 |  | 10.9 | 14.9 | -12.1 | 26.7 |  | 10.3 | 12.7 | -8.3 | 24.8 |
| RH (%) | 76 | 77 | 52 | 100 |  | 63 | 67 | 24 | 91 |  | 55 | 53 | 14 | 89 |  | 55 | 53 | 26 | 92 |  | 35 | 35 | 17 | 70 |  | 46 | 44 | 16 | 87 |
| Solar radiation (W m^-2^) ^b^ | 736 | 775 | 198 | 1145 |  | 735 | 759 | 403 | 1084 |  | 651 | 646 | 386 | 1002 |  | 720 | 717 | 261 | 1135 |  | 787 | 756 | 447 | 1220 |  | 754 | 700 | 420 | 1114 |
| 3-MeTHF-3,4-diols | 0.45 | 0.17 | 0.04 | 2.36 |  | 0.21 | 0.14 | 0.04 | 1.34 |  | 0.60 | 0.23 | nd ^c^ | 2.50 |  | 0.23 | 0.11 | nd | 1.09 |  | 0.29 | 0.18 | 0.04 | 1.83 |  | 0.08 | 0.07 | nd | 0.20 |
| C_5_-alkenetriols | 13.7 | 7.11 | 2.22 | 72.6 |  | 5.61 | 4.81 | 0.63 | 23.7 |  | 12.6 | 4.66 | 0.40 | 55.9 |  | 5.83 | 2.42 | 0.45 | 34.4 |  | 5.51 | 2.67 | 0.63 | 32.4 |  | 1.94 | 1.41 | 0.35 | 5.15 |
| 2-Methylglyceric acid (MGA) | 4.15 | 2.00 | 0.46 | 18.2 |  | 3.25 | 2.42 | 0.25 | 12.9 |  | 5.99 | 4.10 | 0.26 | 22.2 |  | 2.36 | 0.88 | 0.20 | 9.44 |  | 1.24 | 0.98 | 0.27 | 4.83 |  | 0.85 | 0.73 | 0.16 | 3.03 |
| 2-Methyltetrols (MTLs) | 35.2 | 6.11 | 0.93 | 244 |  | 20.2 | 5.82 | 0.85 | 150 |  | 25.1 | 4.97 | 0.54 | 142 |  | 11.4 | 3.17 | 1.18 | 59.0 |  | 15.9 | 10.5 | 2.25 | 51.2 |  | 3.80 | 1.70 | 0.51 | 21.0 |
| Sum of tracers | 53.5 | 16.2 | 3.89 | 337 |  | 29.1 | 14.1 | 3.19 | 188 |  | 44.3 | 14.8 | 1.22 | 222 |  | 19.9 | 6.48 | 1.89 | 101 |  | 22.8 | 15.6 | 3.39 | 78.5 |  | 6.67 | 4.50 | 1.87 | 26.8 |
| MGA/MTLs | 0.37 | 0.35 | 0.04 | 1.12 |  | 0.46 | 0.40 | 0.04 | 1.27 |  | 1.05 | 0.35 | 0.08 | 5.44 |  | 0.32 | 0.23 | 0.08 | 1.53 |  | 0.11 | 0.10 | 0.02 | 0.35 |  | 0.40 | 0.37 | 0.06 | 1.00 |
| SOC (μgC m^-3^) | 0.25 | 0.06 | 0.01 | 1.69 |  | 0.15 | 0.06 | 0.01 | 1.05 |  | 0.20 | 0.08 | 0.01 | 1.06 |  | 0.09 | 0.03 | 0.01 | 0.42 |  | 0.11 | 0.07 | 0.02 | 0.34 |  | 0.03 | 0.02 | 0.01 | 0.14 |
|  | **Fall (October –November 2012)** | | | | | | | | | | | | | | | | | | | | | | | | | | | | |
| Temperature (°C) | -0.9 | -0.4 | -16.0 | 12.0 |  | 0.9 | -1.9 | -11.1 | 13.5 |  | 9.8 | 10.1 | 2.4 | 17.9 |  | 6.9 | 8.1 | 1.2 | 13.0 |  | 4.9 | 5.2 | -3.1 | 15.6 |  | 5.6 | 5.7 | -2.4 | 13.0 |
| RH (%) | 81 | 86 | 64 | 100 |  | 69 | 68 | 58 | 86 |  | 46 | 47 | 27 | 63 |  | 49 | 44 | 33 | 65 |  | 35 | 34 | 32 | 40 |  | 48 | 49 | 36 | 59 |
| Solar radiation (W m^-2^) | 448 | 424 | 198 | 673 |  | 523 | 472 | 440 | 709 |  | 561 | 560 | 434 | 671 |  | 435 | 552 | 448 | 750 |  | 642 | 628 | 515 | 772 |  | 626 | 603 | 554 | 724 |
| 3-MeTHF-3,4-diols | 0.10 | 0.07 | 0.05 | 0.25 |  | 0.07 | 0.06 | nd | 0.12 |  | 0.08 | 0.07 | nd | 0.14 |  | 0.13 | 0.07 | nd | 0.30 |  | 0.64 | 0.46 | 0.15 | 1.83 |  | 0.06 | 0.05 | nd | 0.13 |
| C_5_-alkenetriols | 8.00 | 4.79 | 3.15 | 18.0 |  | 5.89 | 5.23 | 2.85 | 10.2 |  | 2.15 | 1.14 | 0.40 | 6.72 |  | 3.25 | 2.46 | 1.86 | 5.30 |  | 8.66 | 10.6 | 0.86 | 16.1 |  | 2.21 | 1.35 | 0.80 | 5.15 |
| 2-Methylglyceric acid (MGA) | 1.74 | 0.78 | 0.52 | 4.77 |  | 1.55 | 1.70 | 0.36 | 2.57 |  | 5.27 | 0.69 | 0.26 | 14.8 |  | 2.64 | 0.41 | 0.20 | 6.19 |  | 0.47 | 0.44 | 0.32 | 0.65 |  | 0.81 | 0.25 | 0.16 | 3.03 |
| 2-Methyltetrols (MTLs) | 3.43 | 2.34 | 0.93 | 6.74 |  | 2.89 | 2.95 | 1.82 | 3.85 |  | 2.21 | 2.73 | 0.54 | 3.98 |  | 2.90 | 1.80 | 1.34 | 5.93 |  | 11.7 | 11.9 | 4.75 | 20.5 |  | 2.71 | 1.01 | 0.64 | 8.51 |
| Sum of tracers | 13.3 | 10.4 | 6.31 | 29.7 |  | 10.0 | 8.63 | 6.40 | 16.3 |  | 9.72 | 11.5 | 1.22 | 18.8 |  | 8.92 | 4.69 | 3.50 | 17.6 |  | 21.3 | 23.7 | 8.48 | 38.5 |  | 5.78 | 3.96 | 1.87 | 16.8 |
| MGA/MTLs | 0.46 | 0.39 | 0.31 | 0.71 |  | 0.61 | 0.75 | 0.15 | 0.94 |  | 2.01 | 0.48 | 0.17 | 5.44 |  | 0.64 | 0.30 | 0.15 | 1.53 |  | 0.04 | 0.05 | 0.02 | 0.05 |  | 0.27 | 0.34 | 0.10 | 0.36 |
| SOC (μgC m^-3^) | 0.03 | 0.02 | 0.01 | 0.07 |  | 0.03 | 0.02 | 0.02 | 0.04 |  | 0.05 | 0.03 | 0.01 | 0.11 |  | 0.04 | 0.01 | 0.01 | 0.08 |  | 0.08 | 0.08 | 0.03 | 0.13 |  | 0.02 | 0.01 | 0.01 | 0.07 |
|  | **Winter (December 2012- February 2013)** | | | | | | | | | | | | | | | | | | | | | | | | | | | | |
| Temperature (°C) | -22.3 | -20.3 | -29.4 | -18.7 |  | -18.4 | -18.3 | -22.7 | -13.5 |  | -4.3 | -3.6 | -9.1 | -1.5 |  | -4.5 | -4.7 | -8.4 | -1.2 |  | -6.7 | -7.0 | -12.1 | -1.4 |  | -5.0 | -4.5 | -8.3 | -1.2 |
| RH (%) | 76 | 73 | 72 | 88 |  | 63 | 67 | 47 | 76 |  | 51 | 49 | 32 | 78 |  | 59 | 48 | 40 | 92 |  | 48 | 50 | 24 | 70 |  | 42 | 41 | 31 | 53 |
| Solar radiation (W m^-2^) | 432 | 413 | 262 | 635 |  | 479 | 426 | 403 | 659 |  | 458 | 424 | 386 | 653 |  | 435 | 438 | 261 | 612 |  | 559 | 549 | 447 | 706 |  | 510 | 505 | 420 | 602 |
| 3-MeTHF-3,4-diols | 0.48 | 0.26 | 0.06 | 1.14 |  | 0.13 | 0.08 | 0.05 | 0.32 |  | 0.54 | 0.23 | 0.05 | 1.38 |  | 0.11 | 0.09 | 0.05 | 0.22 |  | 0.40 | 0.34 | 0.22 | 0.65 |  | 0.07 | 0.07 | 0.05 | 0.09 |
| C_5_-alkenetriols | 7.76 | 8.52 | 4.06 | 10.9 |  | 4.65 | 5.10 | 2.03 | 6.37 |  | 6.50 | 3.98 | 2.04 | 17.7 |  | 2.19 | 1.98 | 0.96 | 4.83 |  | 12.5 | 7.38 | 6.40 | 32.4 |  | 2.42 | 2.30 | 0.85 | 3.72 |
| 2-Methylglyceric acid (MGA) | 3.84 | 3.49 | 0.65 | 8.29 |  | 2.02 | 1.37 | 0.25 | 5.10 |  | 3.13 | 1.93 | 0.45 | 8.21 |  | 0.46 | 0.44 | 0.20 | 0.85 |  | 1.33 | 1.45 | 0.73 | 1.75 |  | 0.59 | 0.58 | 0.47 | 0.81 |
| 2-Methyltetrols (MTLs) | 6.36 | 3.83 | 1.50 | 13.3 |  | 3.18 | 2.16 | 0.85 | 7.54 |  | 6.22 | 2.49 | 1.24 | 15.3 |  | 2.08 | 1.65 | 1.30 | 3.42 |  | 14.3 | 7.18 | 5.06 | 44.6 |  | 1.12 | 1.05 | 0.51 | 1.73 |
| Sum of tracers | 18.4 | 16.2 | 10.7 | 33.6 |  | 10.0 | 8.70 | 3.19 | 19.3 |  | 16.3 | 8.28 | 3.79 | 39.6 |  | 4.84 | 4.36 | 2.56 | 8.47 |  | 28.2 | 15.6 | 12.9 | 78.5 |  | 4.20 | 4.03 | 1.91 | 6.21 |
| MGA/MTLs | 0.60 | 0.52 | 0.43 | 0.91 |  | 0.55 | 0.59 | 0.30 | 0.72 |  | 0.66 | 0.47 | 0.30 | 1.46 |  | 0.22 | 0.22 | 0.15 | 0.27 |  | 0.16 | 0.13 | 0.03 | 0.35 |  | 0.59 | 0.56 | 0.35 | 0.93 |
| SOC (μgC m^-3^) | 0.07 | 0.05 | 0.01 | 0.14 |  | 0.03 | 0.02 | 0.01 | 0.08 |  | 0.06 | 0.03 | 0.01 | 0.14 |  | 0.02 | 0.01 | 0.01 | 0.03 |  | 0.10 | 0.05 | 0.04 | 0.30 |  | 0.01 | 0.01 | 0.01 | 0.02 |
|  | **Spring (March-May 2013)** | | | | | | | | | | | | | | | | | | | | | | | | | | | | |
| Temperature (°C) | 2.8 | 0.2 | -11.4 | 20.1 |  | 5.7 | 3.3 | -7.6 | 20.0 |  | 14.7 | 13.0 | 5.6 | 23.2 |  | 15.3 | 16.5 | 6.6 | 23.3 |  | 16.1 | 19.3 | 7.4 | 21.5 |  | 14.7 | 17.2 | 5.7 | 19.6 |
| RH (%) | 70 | 72 | 52 | 80 |  | 52 | 51 | 24 | 85 |  | 42 | 40 | 14 | 82 |  | 36 | 32 | 26 | 71 |  | 21 | 21 | 17 | 25 |  | 24 | 22 | 16 | 36 |
| Solar radiation (W m^-2^) | 934 | 918 | 697 | 1138 |  | 936 | 920 | 731 | 1084 |  | 799 | 836 | 639 | 960 |  | 966 | 971 | 793 | 1102 |  | 885 | 900 | 677 | 1056 |  | 929 | 907 | 799 | 1081 |
| 3-MeTHF-3,4-diols | 0.12 | 0.12 | 0.04 | 0.24 |  | 0.17 | 0.15 | 0.06 | 0.36 |  | 0.16 | 0.12 | 0.05 | 0.29 |  | 0.09 | 0.08 | nd | 0.18 |  | 0.10 | 0.10 | 0.04 | 0.17 |  | 0.10 | 0.10 | nd | 0.20 |
| C_5_-alkenetriols | 5.10 | 5.36 | 2.22 | 8.99 |  | 5.27 | 7.19 | 1.32 | 8.07 |  | 3.31 | 3.31 | 1.08 | 6.44 |  | 1.46 | 1.57 | 0.45 | 2.46 |  | 1.00 | 0.95 | 0.63 | 1.54 |  | 1.95 | 1.22 | 0.43 | 4.60 |
| 2-Methylglyceric acid (MGA) | 1.93 | 1.16 | 0.46 | 7.02 |  | 3.53 | 2.77 | 1.07 | 6.90 |  | 3.86 | 3.74 | 0.93 | 8.04 |  | 0.88 | 0.80 | 0.24 | 2.02 |  | 0.80 | 0.91 | 0.27 | 1.27 |  | 0.99 | 0.99 | 0.58 | 1.41 |
| 2-Methyltetrols (MTLs) | 4.83 | 5.00 | 1.14 | 9.84 |  | 14.4 | 5.82 | 0.97 | 63.1 |  | 5.59 | 2.14 | 0.88 | 17.2 |  | 2.76 | 2.39 | 1.18 | 5.77 |  | 5.24 | 4.50 | 2.25 | 10.3 |  | 1.77 | 1.67 | 0.90 | 2.97 |
| Sum of tracers | 12.0 | 12.4 | 3.89 | 19.2 |  | 23.3 | 14.1 | 3.42 | 78.4 |  | 12.9 | 9.92 | 4.90 | 32.0 |  | 5.18 | 4.95 | 1.89 | 9.08 |  | 7.14 | 6.76 | 3.39 | 12.4 |  | 4.81 | 5.02 | 2.52 | 7.47 |
| MGA/MTLs | 0.43 | 0.37 | 0.10 | 1.12 |  | 0.65 | 0.61 | 0.11 | 1.27 |  | 1.71 | 2.16 | 0.09 | 3.30 |  | 0.32 | 0.34 | 0.21 | 0.43 |  | 0.16 | 0.16 | 0.10 | 0.20 |  | 0.60 | 0.57 | 0.39 | 1.00 |
| SOC (μgC m^-3^) | 0.04 | 0.04 | 0.01 | 0.09 |  | 0.12 | 0.06 | 0.01 | 0.45 |  | 0.06 | 0.04 | 0.02 | 0.16 |  | 0.02 | 0.02 | 0.01 | 0.05 |  | 0.04 | 0.03 | 0.02 | 0.07 |  | 0.02 | 0.02 | 0.01 | 0.03 |
|  | **Summer (June-September 2013)** | | | | | | | | | | | | | | | | | | | | | | | | | | | | |
| Temperature (°C) | 19.4 | 20.4 | 11.8 | 25.2 |  | 20.4 | 22.3 | 13.2 | 25.1 |  | 24.4 | 24.3 | 20.7 | 28.2 |  | 21.7 | 22.1 | 16.7 | 26.3 |  | 23.3 | 23.2 | 18.7 | 26.7 |  | 21.0 | 21.0 | 17.0 | 24.8 |
| RH (%) | 77 | 79 | 59 | 89 |  | 70 | 69 | 45 | 91 |  | 74 | 74 | 59 | 89 |  | 72 | 71 | 58 | 81 |  | 38 | 38 | 31 | 47 |  | 67 | 65 | 52 | 87 |
| Solar radiation (W m^-2^) | 972 | 981 | 818 | 1145 |  | 857 | 872 | 540 | 1045 |  | 723 | 754 | 515 | 1002 |  | 818 | 901 | 301 | 1135 |  | 962 | 1004 | 686 | 1220 |  | 864 | 926 | 584 | 1114 |
| 3-MeTHF-3,4-diols | 1.00 | 0.69 | 0.10 | 2.36 |  | 0.38 | 0.23 | 0.06 | 1.34 |  | 1.37 | 1.35 | 0.61 | 2.50 |  | 0.51 | 0.46 | 0.12 | 1.09 |  | 0.19 | 0.18 | 0.09 | 0.27 |  | 0.08 | 0.08 | nd | 0.19 |
| C_5_-alkenetriols | 30.6 | 28.0 | 5.17 | 72.6 |  | 6.45 | 4.01 | 0.63 | 23.7 |  | 31.9 | 30.6 | 10.2 | 55.9 |  | 14.0 | 10.8 | 2.57 | 34.4 |  | 2.25 | 2.28 | 0.77 | 3.73 |  | 1.40 | 1.23 | 0.35 | 4.42 |
| 2-Methylglyceric acid (MGA) | 8.32 | 5.79 | 1.74 | 18.2 |  | 4.61 | 2.82 | 0.95 | 12.9 |  | 10.5 | 8.31 | 3.28 | 22.2 |  | 4.90 | 4.76 | 2.22 | 9.44 |  | 1.85 | 1.47 | 0.45 | 4.83 |  | 0.96 | 1.07 | 0.26 | 1.70 |
| 2-Methyltetrols (MTLs) | 109 | 94.4 | 17.9 | 244 |  | 49.9 | 25.0 | 7.20 | 150 |  | 70.5 | 72.5 | 18.0 | 142 |  | 31.4 | 28.8 | 13.8 | 59.0 |  | 29.1 | 30.7 | 10.7 | 51.2 |  | 8.28 | 5.59 | 1.42 | 21.0 |
| Sum of tracers | 149 | 123 | 25.0 | 337 |  | 61.3 | 33.2 | 10.4 | 188 |  | 114 | 116 | 32.5 | 222 |  | 50.8 | 48.4 | 19.2 | 101 |  | 33.4 | 34.2 | 12.0 | 56.5 |  | 10.7 | 8.05 | 2.20 | 26.8 |
| MGA/MTLs | 0.08 | 0.07 | 0.04 | 0.13 |  | 0.12 | 0.07 | 0.04 | 0.24 |  | 0.15 | 0.15 | 0.08 | 0.21 |  | 0.18 | 0.14 | 0.08 | 0.48 |  | 0.06 | 0.04 | 0.03 | 0.14 |  | 0.18 | 0.14 | 0.06 | 0.40 |
| SOC (μgC m^-3^) | 0.76 | 0.65 | 0.13 | 1.69 |  | 0.35 | 0.19 | 0.06 | 1.05 |  | 0.52 | 0.54 | 0.14 | 1.06 |  | 0.23 | 0.22 | 0.11 | 0.42 |  | 0.20 | 0.21 | 0.07 | 0.34 |  | 0.06 | 0.04 | 0.01 | 0.14 |

**Table S1 Summary of SOA data at 12 sites in China (C**ontinued)

|  | **East China** | | | | | | | | | | | | | |  | **Southwest China** | | | | | | | | |  | **South China** | | | |
| --- | --- | --- | --- | --- | --- | --- | --- | --- | --- | --- | --- | --- | --- | --- | --- | --- | --- | --- | --- | --- | --- | --- | --- | --- | --- | --- | --- | --- | --- |
|  | **Hefei (HF)** | | | |  | **Wuxi (WX)** | | | |  | **Qianyanzhou (QYZ)** | | | |  | **Kunming (KM)** | | | |  | **Xishuangbanna (BN)** | | | |  | **Sanya (SY)** | | | |
|  | Urban | | | |  | Sub-urban | | | |  | Rural | | | |  | Urban | | | |  | Rural | | | |  | Sub-urban | | | |
|  | mean | median | min | max |  | mean | median | min | max |  | mean | median | min | max |  | mean | median | min | max |  | mean | median | min | max |  | mean | median | min | max |
|  | **Whole year (October 2012-September 2013)** | | | | | | | | | | | | | | | | | | | | | | | | | | | | |
| Temperature (°C) | 17.0 | 18.9 | 0.9 | 32.5 |  | 17.3 | 19.1 | 0.9 | 33.5 |  | 20.0 | 21.2 | 3.5 | 33.7 |  | 16.6 | 16.9 | 9.3 | 22.8 |  | 23.0 | 23.3 | 18.0 | 26.4 |  | 23.1 | 23.5 | 17.4 | 26.8 |
| RH (%) | 75 | 74 | 51 | 97 |  | 67 | 65 | 45 | 90 |  | 77 | 78 | 48 | 95 |  | 66 | 66 | 43 | 88 |  | 76 | 79 | 56 | 83 |  | 91 | 93 | 79 | 100 |
| Solar radiation (W m^-2^) | 736 | 793 | 105 | 1202 |  | 730 | 777 | 81 | 1224 |  | 789 | 860 | 119 | 1423 |  | 970 | 931 | 637 | 1304 |  | 988 | 969 | 578 | 1335 |  | 1052 | 1080 | 594 | 1374 |
| 3-MeTHF-3,4-diols | 0.47 | 0.18 | 0.05 | 2.14 |  | 0.36 | 0.16 | 0.05 | 2.01 |  | 0.70 | 0.29 | 0.05 | 2.48 |  | 0.44 | 0.42 | 0.13 | 1.09 |  | 0.41 | 0.24 | 0.05 | 2.24 |  | 0.13 | 0.10 | nd | 0.36 |
| C_5_-alkenetriols | 13.2 | 4.24 | 1.12 | 86.3 |  | 15.7 | 3.16 | 0.44 | 154 |  | 24.0 | 6.82 | 0.77 | 117 |  | 18.9 | 11.6 | 2.57 | 105 |  | 23.7 | 9.64 | 0.24 | 178 |  | 3.08 | 1.60 | 0.25 | 12.5 |
| 2-Methylglyceric acid (MGA) | 4.24 | 2.64 | 0.27 | 15.0 |  | 4.65 | 2.19 | 0.51 | 22.1 |  | 4.73 | 3.61 | 0.77 | 16.7 |  | 5.96 | 5.64 | 2.19 | 18.7 |  | 3.51 | 2.41 | 0.26 | 12.8 |  | 2.93 | 2.16 | 0.35 | 11.0 |
| 2-Methyltetrols (MTLs) | 40.3 | 8.59 | 0.85 | 174 |  | 30.5 | 8.84 | 0.89 | 182 |  | 62.0 | 39.2 | 1.94 | 226 |  | 82.7 | 67.6 | 20.1 | 191 |  | 94.6 | 80.4 | 5.41 | 342 |  | 24.9 | 19.1 | 5.23 | 69.9 |
| Sum of tracers | 58.2 | 22.4 | 3.12 | 277 |  | 51.2 | 14.1 | 3.31 | 360 |  | 91.5 | 56.7 | 4.09 | 358 |  | 108 | 87.4 | 31.6 | 306 |  | 122 | 102 | 6.42 | 529 |  | 31.0 | 22.6 | 5.97 | 85.9 |
| MGA/MTLs | 0.37 | 0.18 | 0.04 | 1.23 |  | 0.35 | 0.36 | 0.04 | 0.74 |  | 0.20 | 0.15 | 0.02 | 0.76 |  | 0.09 | 0.08 | 0.02 | 0.28 |  | 0.05 | 0.03 | 0.00 | 0.16 |  | 0.12 | 0.12 | 0.04 | 0.31 |
| SOC | 0.29 | 0.07 | 0.01 | 1.22 |  | 0.23 | 0.07 | 0.01 | 1.32 |  | 0.43 | 0.27 | 0.02 | 1.54 |  | 0.57 | 0.48 | 0.17 | 1.29 |  | 0.63 | 0.53 | 0.04 | 2.25 |  | 0.18 | 0.14 | 0.04 | 0.47 |
|  | **Fall (October –November 2012)** | | | | | | | | | | | | | | | | | | | | | | | | | | | | |
| Temperature (°C) | 13.9 | 14.8 | 6.6 | 20.1 |  | 14.4 | 15.2 | 6.7 | 20.8 |  | 17.4 | 18.9 | 10.8 | 23.6 |  | 15.5 | 15.6 | 13.3 | 18.6 |  | 23.0 | 23.4 | 21.9 | 24.1 |  | 23.2 | 23.0 | 22.7 | 24.2 |
| RH (%) | 76 | 75 | 64 | 89 |  | 67 | 71 | 54 | 78 |  | 78 | 79 | 62 | 94 |  | 74 | 71 | 67 | 86 |  | 81 | 82 | 77 | 83 |  | 87 | 84 | 82 | 98 |
| Solar radiation (W m^-2^) | 566 | 574 | 298 | 822 |  | 644 | 646 | 512 | 818 |  | 766 | 837 | 332 | 1038 |  | 868 | 923 | 637 | 968 |  | 1029 | 1006 | 840 | 1188 |  | 1090 | 1080 | 1065 | 1135 |
| 3-MeTHF-3,4-diols | 0.14 | 0.13 | 0.11 | 0.18 |  | 0.10 | 0.10 | 0.08 | 0.11 |  | 0.93 | 0.56 | 0.18 | 2.07 |  | 0.55 | 0.50 | 0.31 | 0.87 |  | 0.88 | 0.63 | 0.37 | 2.24 |  | 0.15 | 0.10 | 0.08 | 0.36 |
| C_5_-alkenetriols | 6.05 | 3.53 | 1.57 | 15.6 |  | 2.75 | 3.06 | 1.95 | 3.25 |  | 25.8 | 21.5 | 3.64 | 70.4 |  | 31.0 | 29.6 | 19.3 | 43.5 |  | 67.1 | 37.2 | 22.0 | 178 |  | 4.94 | 2.88 | 0.84 | 12.5 |
| 2-Methylglyceric acid (MGA) | 1.27 | 0.99 | 0.27 | 2.81 |  | 1.15 | 1.00 | 0.63 | 1.81 |  | 6.40 | 4.07 | 3.62 | 12.8 |  | 4.72 | 4.86 | 2.36 | 7.31 |  | 4.82 | 3.58 | 3.07 | 7.18 |  | 1.84 | 1.46 | 0.74 | 3.14 |
| 2-Methyltetrols (MTLs) | 9.36 | 7.04 | 2.31 | 21.0 |  | 4.08 | 2.21 | 1.28 | 8.75 |  | 41.1 | 34.3 | 11.2 | 82.6 |  | 110 | 109 | 41.0 | 167 |  | 177 | 133 | 119 | 342 |  | 36.1 | 28.4 | 17.6 | 69.9 |
| Sum of tracers | 16.8 | 17.1 | 7.43 | 25.6 |  | 8.08 | 6.58 | 5.06 | 12.6 |  | 74.1 | 61.9 | 19.5 | 167 |  | 146 | 156 | 68.1 | 204 |  | 250 | 194 | 155 | 529 |  | 43.1 | 32.8 | 19.9 | 85.9 |
| MGA/MTLs | 0.18 | 0.13 | 0.04 | 0.41 |  | 0.38 | 0.45 | 0.21 | 0.49 |  | 0.18 | 0.16 | 0.09 | 0.32 |  | 0.05 | 0.04 | 0.02 | 0.12 |  | 0.03 | 0.03 | 0.02 | 0.04 |  | 0.05 | 0.04 | 0.04 | 0.07 |
| SOC | 0.07 | 0.05 | 0.02 | 0.15 |  | 0.03 | 0.02 | 0.01 | 0.07 |  | 0.31 | 0.25 | 0.10 | 0.62 |  | 0.74 | 0.72 | 0.30 | 1.12 |  | 1.18 | 0.88 | 0.79 | 2.25 |  | 0.24 | 0.19 | 0.12 | 0.47 |
|  | **Winter (December 2012- February 2013)** | | | | | | | | | | | | | | | | | | | | | | | | | | | | |
| Temperature (°C) | 2.6 | 2.3 | 0.9 | 5.4 |  | 3.6 | 3.4 | 0.9 | 6.9 |  | 8.5 | 7.4 | 3.5 | 18.7 |  | 11.0 | 10.2 | 9.3 | 14.8 |  | 19.3 | 18.9 | 18.0 | 22.3 |  | 19.8 | 20.2 | 17.4 | 21.7 |
| RH (%) | 79 | 82 | 51 | 95 |  | 75 | 79 | 56 | 87 |  | 87 | 88 | 79 | 94 |  | 58 | 57 | 50 | 67 |  | 74 | 74 | 66 | 80 |  | 87 | 87 | 79 | 93 |
| Solar radiation (W m^-2^) | 345 | 432 | 105 | 518 |  | 379 | 468 | 81 | 613 |  | 483 | 539 | 119 | 695 |  | 831 | 782 | 752 | 1013 |  | 832 | 831 | 742 | 972 |  | 977 | 943 | 893 | 1192 |
| 3-MeTHF-3,4-diols | 0.31 | 0.11 | 0.05 | 1.13 |  | 0.18 | 0.16 | 0.05 | 0.36 |  | 0.09 | 0.08 | 0.05 | 0.12 |  | 0.30 | 0.21 | 0.13 | 0.51 |  | 0.52 | 0.60 | 0.05 | 1.13 |  | 0.14 | 0.13 | 0.09 | 0.22 |
| C_5_-alkenetriols | 5.50 | 4.24 | 1.12 | 10.8 |  | 3.80 | 3.88 | 1.94 | 5.50 |  | 2.81 | 2.61 | 0.77 | 6.09 |  | 12.1 | 11.6 | 5.84 | 18.5 |  | 19.5 | 24.0 | 0.24 | 35.1 |  | 2.28 | 1.90 | 0.64 | 5.84 |
| 2-Methylglyceric acid (MGA) | 1.67 | 1.52 | 0.92 | 3.02 |  | 1.78 | 1.69 | 0.51 | 3.22 |  | 1.42 | 1.27 | 0.77 | 2.40 |  | 5.69 | 5.60 | 3.80 | 6.89 |  | 5.36 | 5.04 | 0.72 | 12.8 |  | 2.79 | 2.58 | 1.95 | 4.37 |
| 2-Methyltetrols (MTLs) | 4.90 | 2.74 | 0.85 | 16.8 |  | 3.30 | 3.27 | 0.89 | 5.75 |  | 3.84 | 3.44 | 1.94 | 6.33 |  | 52.9 | 43.9 | 20.1 | 101 |  | 84.8 | 94.3 | 5.41 | 172 |  | 18.9 | 18.3 | 10.4 | 30.9 |
| Sum of tracers | 12.4 | 8.89 | 3.12 | 31.0 |  | 9.06 | 9.05 | 4.05 | 14.1 |  | 8.16 | 7.93 | 4.09 | 14.9 |  | 71.0 | 63.2 | 31.6 | 119 |  | 110 | 127 | 6.42 | 204 |  | 24.1 | 22.6 | 13.2 | 41.4 |
| MGA/MTLs | 0.68 | 0.64 | 0.18 | 1.08 |  | 0.57 | 0.59 | 0.45 | 0.66 |  | 0.38 | 0.40 | 0.23 | 0.50 |  | 0.15 | 0.09 | 0.06 | 0.28 |  | 0.08 | 0.08 | 0.03 | 0.13 |  | 0.15 | 0.15 | 0.12 | 0.19 |
| SOC | 0.04 | 0.03 | 0.01 | 0.13 |  | 0.03 | 0.03 | 0.01 | 0.05 |  | 0.03 | 0.03 | 0.02 | 0.06 |  | 0.38 | 0.31 | 0.17 | 0.69 |  | 0.58 | 0.64 | 0.04 | 1.15 |  | 0.14 | 0.13 | 0.08 | 0.23 |
|  | **Spring (March-May 2013)** | | | | | | | | | | | | | | | | | | | | | | | | | | | | |
| Temperature (°C) | 19.3 | 19.4 | 12.7 | 26.3 |  | 18.8 | 19.5 | 11.1 | 25.7 |  | 21.4 | 22.7 | 12.4 | 29.0 |  | 18.3 | 17.6 | 13.6 | 22.8 |  | 23.9 | 23.2 | 20.8 | 26.4 |  | 23.6 | 23.7 | 17.4 | 26.8 |
| RH (%) | 69 | 63 | 57 | 89 |  | 59 | 56 | 45 | 75 |  | 77 | 75 | 60 | 94 |  | 55 | 55 | 43 | 64 |  | 69 | 70 | 56 | 80 |  | 92 | 94 | 83 | 95 |
| Solar radiation (W m^-2^) | 878 | 920 | 695 | 1038 |  | 912 | 933 | 738 | 1027 |  | 862 | 903 | 332 | 1136 |  | 1134 | 1133 | 888 | 1287 |  | 996 | 975 | 848 | 1221 |  | 1074 | 1083 | 775 | 1344 |
| 3-MeTHF-3,4-diols | 0.11 | 0.07 | 0.05 | 0.25 |  | 0.19 | 0.19 | 0.05 | 0.49 |  | 0.42 | 0.17 | 0.09 | 1.20 |  | 0.37 | 0.41 | 0.14 | 0.58 |  | 0.24 | 0.20 | 0.11 | 0.53 |  | 0.11 | 0.08 | 0.05 | 0.26 |
| C_5_-alkenetriols | 2.22 | 1.63 | 1.39 | 4.23 |  | 2.84 | 3.00 | 0.44 | 6.39 |  | 9.12 | 5.51 | 2.08 | 26.6 |  | 8.42 | 9.26 | 4.05 | 12.3 |  | 7.87 | 5.97 | 1.20 | 25.1 |  | 3.02 | 1.60 | 1.24 | 7.50 |
| 2-Methylglyceric acid (MGA) | 2.16 | 2.11 | 1.20 | 3.24 |  | 3.60 | 2.59 | 0.58 | 8.58 |  | 3.77 | 3.61 | 1.58 | 6.53 |  | 7.20 | 6.95 | 2.19 | 18.7 |  | 2.92 | 1.11 | 0.26 | 10.6 |  | 2.94 | 2.21 | 0.64 | 6.90 |
| 2-Methyltetrols (MTLs) | 12.9 | 5.25 | 1.27 | 39.8 |  | 10.0 | 8.93 | 2.03 | 27.8 |  | 41.1 | 14.8 | 4.77 | 102 |  | 67.9 | 63.4 | 36.8 | 123 |  | 68.6 | 67.4 | 5.67 | 146 |  | 21.4 | 13.6 | 5.23 | 56.0 |
| Sum of tracers | 17.4 | 8.87 | 4.28 | 47.6 |  | 16.6 | 14.2 | 3.31 | 43.3 |  | 54.4 | 23.1 | 9.30 | 134 |  | 83.9 | 80.4 | 53.9 | 151 |  | 79.6 | 86.7 | 7.28 | 155 |  | 27.5 | 18.0 | 8.52 | 70.6 |
| MGA/MTLs | 0.56 | 0.47 | 0.08 | 1.23 |  | 0.42 | 0.40 | 0.12 | 0.74 |  | 0.23 | 0.18 | 0.04 | 0.76 |  | 0.10 | 0.11 | 0.04 | 0.19 |  | 0.05 | 0.03 | 0.00 | 0.16 |  | 0.16 | 0.13 | 0.08 | 0.31 |
| SOC | 0.10 | 0.05 | 0.02 | 0.28 |  | 0.09 | 0.08 | 0.02 | 0.24 |  | 0.29 | 0.11 | 0.05 | 0.69 |  | 0.48 | 0.45 | 0.28 | 0.92 |  | 0.46 | 0.50 | 0.04 | 0.95 |  | 0.16 | 0.10 | 0.04 | 0.41 |
|  | **Summer (June-September 2013)** | | | | | | | | | | | | | | | | | | | | | | | | | | | | |
| Temperature (°C) | 27.7 | 28.4 | 20.9 | 32.5 |  | 28.1 | 27.6 | 21.8 | 33.5 |  | 29.2 | 30.2 | 22.0 | 33.7 |  | 20.0 | 20.0 | 17.0 | 22.3 |  | 25.2 | 25.6 | 23.2 | 25.7 |  | 25.2 | 25.5 | 23.9 | 26.2 |
| RH (%) | 77 | 81 | 56 | 97 |  | 69 | 64 | 58 | 90 |  | 67 | 67 | 48 | 95 |  | 75 | 75 | 63 | 88 |  | 79 | 80 | 76 | 82 |  | 95 | 94 | 92 | 100 |
| Solar radiation (W m^-2^) | 1012 | 1046 | 645 | 1202 |  | 887 | 933 | 241 | 1224 |  | 969 | 1032 | 248 | 1423 |  | 996 | 1003 | 715 | 1304 |  | 1073 | 1118 | 578 | 1335 |  | 1064 | 1125 | 594 | 1374 |
| 3-MeTHF-3,4-diols | 1.06 | 0.81 | 0.44 | 2.14 |  | 0.71 | 0.22 | 0.14 | 2.01 |  | 1.28 | 1.20 | 0.23 | 2.48 |  | 0.53 | 0.48 | 0.15 | 1.09 |  | 0.17 | 0.13 | 0.07 | 0.53 |  | 0.10 | 0.09 | nd | 0.23 |
| C_5_-alkenetriols | 31.6 | 19.2 | 7.89 | 86.3 |  | 37.8 | 4.11 | 1.70 | 154 |  | 52.0 | 42.5 | 6.00 | 117 |  | 24.7 | 9.99 | 2.57 | 105 |  | 13.7 | 4.11 | 1.09 | 45.3 |  | 2.58 | 1.34 | 0.25 | 8.66 |
| 2-Methylglyceric acid (MGA) | 9.76 | 9.97 | 4.36 | 15.0 |  | 8.32 | 3.74 | 0.90 | 22.1 |  | 7.00 | 5.58 | 0.99 | 16.7 |  | 5.83 | 5.17 | 3.02 | 10.5 |  | 1.82 | 1.63 | 0.27 | 3.99 |  | 3.69 | 2.68 | 0.35 | 11.0 |
| 2-Methyltetrols (MTLs) | 109 | 113 | 44.2 | 174 |  | 72.0 | 27.2 | 6.58 | 182 |  | 137 | 144 | 44.3 | 226 |  | 97.2 | 88.3 | 34.1 | 191 |  | 73.0 | 64.5 | 26.9 | 134 |  | 25.3 | 24.8 | 5.34 | 47.1 |
| Sum of tracers | 151 | 142 | 56.9 | 277 |  | 119 | 33.0 | 11.7 | 360 |  | 197 | 193 | 51.5 | 358 |  | 128 | 103 | 39.8 | 306 |  | 88.7 | 69.6 | 28.4 | 180 |  | 31.7 | 29.1 | 5.97 | 67.0 |
| MGA/MTLs | 0.11 | 0.09 | 0.06 | 0.20 |  | 0.15 | 0.12 | 0.04 | 0.45 |  | 0.05 | 0.04 | 0.02 | 0.09 |  | 0.07 | 0.06 | 0.04 | 0.12 |  | 0.02 | 0.03 | 0.01 | 0.04 |  | 0.12 | 0.10 | 0.04 | 0.23 |
| SOC | 0.77 | 0.79 | 0.31 | 1.22 |  | 0.52 | 0.19 | 0.06 | 1.32 |  | 0.93 | 0.96 | 0.29 | 1.54 |  | 0.66 | 0.61 | 0.24 | 1.29 |  | 0.48 | 0.43 | 0.18 | 0.89 |  | 0.19 | 0.18 | 0.04 | 0.37 |

^a^ Site abbreviation is in brackets. ^b^ Solar radiation is daily maximum during each sampling episode. ^c^ “nd” means not detected.

**Table S2 Information of s**ampling sites

| **Sites** | **Temperate Zones** | **Latitude**  **(ºN)** | **Longitude**  **(ºE)** | **Sampling duration** |
| --- | --- | --- | --- | --- |
| Hailun (HL) | Sub-humid temperature zone in Heilongjiang Province | 47.43 | 126.63 | biweekly 48-hr |
| Tongyu (TYU) | Humid temperature zone in Jilin Province | 44.42 | 122.87 | biweekly 48-hr |
| Beijing (BJ) | Sub-humid warm temperate zone | 39.97 | 116.37 | biweekly 48-hr |
| Taiyuan (TY) | Sub-humid warm temperate zone in Shanxi Province | 37.87 | 112.53 | biweekly 48-hr |
| Dunhuang (DH) | Arid warm temperate zone in Gansu Province | 40.13 | 94.71 | biweekly 48-hr |
| Shapotou (SPT) | Arid temperate zone in Ningxia Province | 37.45 | 104.95 | biweekly 48-hr |
| Hefei (HF) | Humid sub-tropic zone in Anhui Province | 31.86 | 117.27 | biweekly 48-hr |
| Wuxi (WX) | Humid sub-tropic zone in Jiangsu Province | 31.50 | 120.35 | biweekly 48-hr |
| Qianyanzhou (QYZ) | Humid sub-tropic zone in Jiangxi Province | 26.75 | 115.07 | biweekly 48-hr |
| Kunming (KM) | Humid sub-tropic zone in Yunnan Province | 25.04 | 102.73 | biweekly 48-hr |
| Xishuangbanna (BN) | Humid tropic zone in Yunnan Province | 21.90 | 101.27 | biweekly 48-hr |
| Sanya (SY) | Humid tropic zone in Hainan Island | 18.22 | 109.47 | biweekly 48-hr |


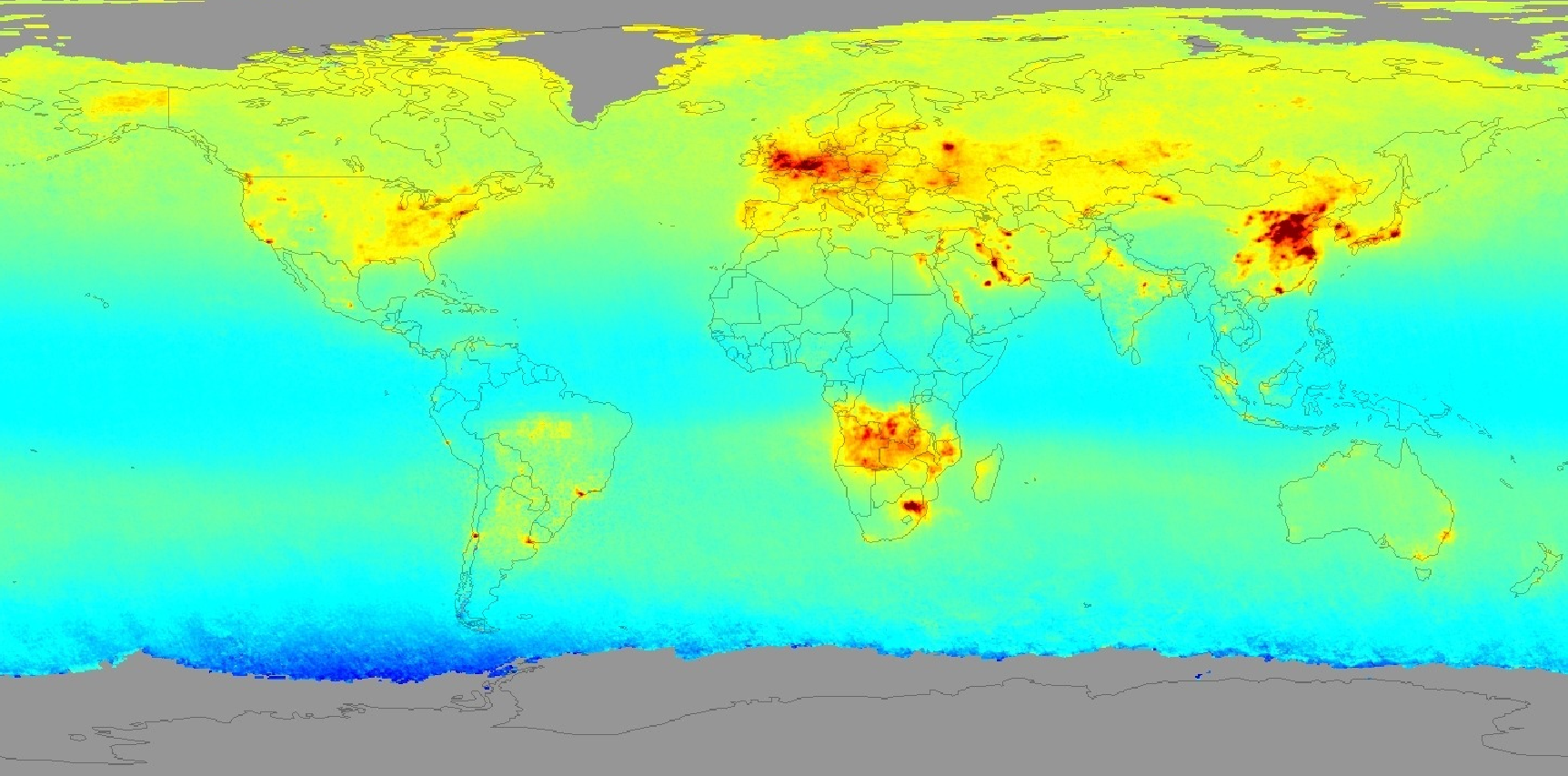


Figure S1 Global total NO_2_ vertical column densities in August 2013. Picture is downloaded from NASA website (http://avdc.gsfc.nasa.gov/)

Figure S2 Correlations between 3-MeTHF-3,4-diol isomers. k is the slope of linear correlation. All correlations are significant at p<0.01. Circle, triangle and square represent urban, sub-urban and rural sites, respectively.

Figure S3 Chromatogram of 3-MeTHF-3,4-diol isomers (*m/z* 262) in SY and DH samples.

Figure S4 Correlations of 3-MeTHF-3,4-diols with C_5_-alkenetriols and 2-methyltetrols at DH

Figure S5 Correlations between SOA_I_ tracers and biogenic emission (C_L_* C_T_) during whole year at 12 sites. Significant correlations (p<0.01) in Northeast China (a-b), North China (c-d) and East China (e-g). Poor correlations (p>0.05) in South China (h), Northwest China (i-j) and Southwest China (k-l)
